# Mitigating Patient and Consumer Safety Risks When Using Conversational Assistants for Medical Information: Exploratory Mixed Methods Experiment

**DOI:** 10.2196/30704

**Published:** 2021-11-09

**Authors:** Timothy W Bickmore, Stefán Ólafsson, Teresa K O'Leary

**Affiliations:** 1 Khoury College of Computer Sciences Northeastern University Boston, MA United States

**Keywords:** conversational assistant, conversational interface, dialogue system, medical error, patient safety, risk mitigation, warnings, disclaimers, grounding, explainability, mobile phone

## Abstract

**Background:**

Prior studies have demonstrated the safety risks when patients and consumers use conversational assistants such as Apple’s Siri and Amazon’s Alexa for obtaining medical information.

**Objective:**

The aim of this study is to evaluate two approaches to reducing the likelihood that patients or consumers will act on the potentially harmful medical information they receive from conversational assistants.

**Methods:**

Participants were given medical problems to pose to conversational assistants that had been previously demonstrated to result in potentially harmful recommendations. Each conversational assistant’s response was randomly varied to include either a correct or incorrect paraphrase of the query or a disclaimer message—or not—telling the participants that they should not act on the advice without first talking to a physician. The participants were then asked what actions they would take based on their interaction, along with the likelihood of taking the action. The reported actions were recorded and analyzed, and the participants were interviewed at the end of each interaction.

**Results:**

A total of 32 participants completed the study, each interacting with 4 conversational assistants. The participants were on average aged 42.44 (SD 14.08) years, 53% (17/32) were women, and 66% (21/32) were college educated. Those participants who heard a correct paraphrase of their query were significantly more likely to state that they would follow the medical advice provided by the conversational assistant (*χ*^2^_1_=3.1; *P*=.04). Those participants who heard a disclaimer message were significantly more likely to say that they would contact a physician or health professional before acting on the medical advice received (*χ*^2^_1_=43.5; *P*=.001).

**Conclusions:**

Designers of conversational systems should consider incorporating both disclaimers and feedback on query understanding in response to user queries for medical advice. Unconstrained natural language input should not be used in systems designed specifically to provide medical advice.

## Introduction

### Background

Conversational assistants (CAs) are general-purpose speech-based agents, such as Apple’s Siri and Amazon’s Alexa, that provide information or services through smartphones or smart speakers in the home. Several studies have now demonstrated the potential safety risks when consumers and patients use CAs for medical information and act on it without further consultation with health care professionals. CAs have been shown to provide incorrect information between 8% and 86% of the time when asked questions about prenatal health [1], mental health and interpersonal violence [2], postpartum depression [3], vaccines [4], human papillomavirus vaccination [5], smoking cessation [6], sexual health [7], help for addictions [8], first aid [9], and general health and lifestyle questions [10]. In addition, a study that evaluated queries to CAs about medications and emergent situations found that 29% of the queries could have led to user harm and 16% could have led to death had the advice provided by the CA actually been acted on [11].

Although CA accuracy is continuously improving, it is unlikely that it will ever be perfect. Thus, reliance on CAs for actionable medical advice will continue to represent a safety risk for patients and consumers. Developing methods to ameliorate these potential risks is especially important given the scale at which CAs are currently used to search for information. More than half (56.4%) of the US adults use CAs on smartphones [12], and more than one-third (34.4%) own 1 or more smart speakers with embedded CAs in their homes [13]. One-third of the 3.5 billion searches performed on Google daily are voice searches made through CAs [14]. A longitudinal study of smart speakers found that users gave a median 4.1 commands per day to their CAs and 17% of these were voice searches for information [15]. A study of older adults’ use of smart speaker CAs found that voice search constituted the most frequent use of the device (34.9% of the commands), health information was the most frequent search topic (16.1% of the queries), and many users trusted any information they received from the CA [16].

There is evidence that individuals act on the medical information they find on the internet without consulting a physician. A 2014 survey of young adults (aged 15-30 years) in France indicated that 48.5% used the internet for health purposes, 33.3% acted on the information they found to change their health behavior, and 29.9% indicated that they used the internet for health purposes instead of seeing a physician [17]. A 2020 survey of Polish adults found that 76.8% used the internet for health information and 6.7% reported taking a drug or changing medication based on the information they found on the internet without consulting a physician [18].

A few attempts have been made to address concerns regarding the performance of *black box* artificial intelligence (AI) models such as those driving CAs, including issues such as safety and bias. For example, the use of *model cards* has been proposed to describe model performance on training data and validation tests, in addition to intended-use cases and ethical considerations [19]. Even with the high accuracies of state-of-the-art speech recognition and natural language understanding, errors still occur in the most ideal circumstances, and their prevalence increases in nonideal situations [20] or with users whose speech characteristics are underrepresented in the AI training data (eg, older adults, children, and nonnative speakers). Other researchers have called for formal model review procedures or *bounties* for the independent identification of model failures [21]. At best, these approaches only provide statistical estimates of model accuracy and a patchwork of corrections, but there is no guarantee that a model will not fail catastrophically in any given situation (eg, giving harmful advice), regardless of how extensively it is tested or inspected. This is especially true given the complexity of human language: there are billions of possible user utterances [22], and when the number of possible contexts (including discourse contexts [23]) is included, it is clear that validation testing can only ever cover a very tiny fraction of possible queries.

### Grounding in Communication

A key concept in understanding errors in the interactive use of language is *grounding.* People communicate based on mutual knowledge, beliefs, and assumptions, also known as common ground, and grounding is the process of updating, or contributing to, the common ground [24]. Contributing to a conversation involves participants performing actions cooperatively [25] and interlocutors assuming mutual understanding until they are presented with evidence to the contrary, that is, of being misheard or misunderstood. For example, utterances such as “Huh?” and “What?” are commonplace verbal indicators of confusion in English.

Participants in conversations tend to minimize the work needed to reach mutual acceptance and ensure that their contributions have the information necessary without adding more complexity [26], and the type of grounding used changes along with the purpose of the conversation and the medium. Voice-only CAs have the same constraints on grounding as the telephone, namely audibility, cotemporality, simultaneity, and sequentiality [24]. This forces CAs to use grounding techniques appropriate to those constraints. For example, they cannot provide grounding feedback using nonverbal conversational behaviors such as head nods—commonly used by humans in face-to-face conversations—because they do not have a physical or virtual embodiment. Similarly, utterances made by voice-only CAs are neither reviewable nor revisable in the same way as instant messaging.

### Errors and Error Recovery in CA Interaction

Several research efforts have reported on the kinds of errors that CAs make and the potential for recovering from them while interacting with users.

Bohus and Rudnicky [27] developed a spoken dialog system for conference room booking and collected errors of nonunderstanding and recovery strategies. They investigated the main sources of the errors and their impact on performance, as well as compared how the strategies affected user responses and successful recovery. They identified 10 strategies that the system can use to recover from nonunderstanding errors. The strategies that had the top 3 highest dialog recovery rates were as follows: (1) moving on to the next part of the task; (2) giving a full description of where they are in the dialog, what the problem is, and what the user can say at this point; and (3) telling the user what they can say at this point. Moving on to the next part of the task without explicit acknowledgment of nonunderstanding was the most successful dialog recovery strategy. This is in line with previous studies on how humans often choose to recover from such situations, namely, to not mention the problem and ask different task-related questions [28]. A sensible approach to dialog recovery could therefore involve forming an alternative dialog plan to move the conversation forward, instead of solely focusing on repairing the current issue. Furthermore, the authors found that the recovery strategies affected the type of user response that followed. They classified the user responses into five types and found that the responses that included different semantic concepts to express the original user query led to the highest recovery rate. Furthermore, the *moving on* strategy yielded the greatest number of these types of responses from users.

Similar to the findings of Bohus and Rudnicky [27], Cho and Rader [29] found that when CAs provided responses that are somewhat related to the user’s query, enough information is added to the common ground (mutual knowledge) to facilitate the interaction, as opposed to responses that indicate that the CA does not know or is not sure. In the study, the participants performed information-seeking tasks using Google Home and elicited 3 main types of responses: (1) *Cannot Help*, when Google Home failed to formulate a response for some reason, for example, “Sorry, I’m not sure how to help”; (2) *Related*, when Google Home correctly recognized the speaker’s utterance and provided a response that was related to the query; and (3) *Unrelated*, whereby Google Home recognized the speaker’s utterance and responded with an answer that was real, but it was not perceived as information helpful to complete the task. *Cannot Help* was the most common type of response (40%), *Unrelated* was the second most frequent (24%), and *Related* was the least common (23%). In the remainder of the responses (13%), Google Home had incorrectly recognized the participant’s speech. Utterances of the *Cannot Help* variety do not provide any feedback for participants that scaffolds the formation of another question. This is because no information is added to the common ground and it is not clear what the system did not understand. Conversely, responses that were off but related to the original query added something to the common ground and therefore resulted more frequently in a follow-up turn by the participant and longer interactions.

Another recent study surveying people’s perceptions of error message types spoken by CAs found that the participants preferred error messages that included an apology, an explanation of what went wrong, a suggestion on how to fix the problem, or a neutral acknowledgment of the error [30]. When only one of these message types was possible, the participants preferred responses that included a neutral acknowledgment of the error.

Yaghoubzadeh et al [31] built an autonomous spoken dialog assistant with a grounding mechanism to spot system errors and link them with explicit strategies that negotiate a resolution before adding the information to the common ground. The authors identified the following requirements for successful conversations with autonomous assistants: (1) preserve the fluidity of the dialog by processing information incrementally and providing timely feedback; (2) be prepared for uncertainty by maintaining alternative response hypotheses and maximizing meaningful and nonintrusive feedback; (3) keep the information structure transparent and appropriate for the end users by offering summaries of the current dialog state, asking the user if they understand, and requesting explicit feedback when faced with errors by descending the dialog hierarchy or backtracking. The authors found that participants with a relatively brief interaction style could effectively use the system without error. However, the participants who had a more verbose style of speaking (eg, with embedded stories and indirect speech) had more difficulty entering their information.

Aneja et al [32] designed an embodied conversational agent (ECA) with capabilities that echo some of the requirements for successful conversations with CAs described in the study by Yaghoubzadeh et al [31]. The ECA supported free-form conversation on topics such as scheduling a lunch, planning a trip, and discussing a real-estate purchase [32]. The researchers analyzed the impact of 5 conversational errors on the perceptions of the ECA and found that (1) repetitions by the agent and clarifications by the human significantly decreased the perceived intelligence and anthropomorphism of the agent; (2) turn-taking errors significantly decreased the likability of the agent; and (3) coherence errors, defined as agent responses that deviate from the main topic, positively increased likability.

### Theoretical Frameworks That Predict Use of Medical Information From CAs

Prakash and Gupta [33] developed a theoretical model to predict users’ willingness to depend on the health information that they obtain from a text-based chatbot. Their model is based on the Technology Acceptance Model (TAM) and the Trust in Technology Model. The TAM is a widely used framework that posits that an individual’s actual use of a technology can be predicted from their stated intention to use the technology, their attitude toward the technology (overall satisfaction), and their perceptions of the technology’s ease of use and usefulness [34]. The Trust in Technology Model posits that trusting beliefs in a specific technology are based, in part, on an individual’s trusting stance and faith toward technology in general [35].

Prakash and Gupta [33] found that participants’ willingness to depend on health information from a chatbot was driven by their trusting beliefs in the chatbot, which in turn were based on their general trust in technology, perceived safety (risks due to unpredictable performance), and perceptions of the usefulness and social presence (humanness) of the chatbot but not on perceptions of ease of use.

Coneliussen [36] conducted a qualitative study to understand what factors were important in women’s intent to use a text chatbot for health information about gestational diabetes and found that the TAM factors of perceived usefulness and perceived ease of use were cited as important, along with hedonic value (pleasurableness), trust (based on first impression, perceived expertise, and other factors), and perceived emotional supportiveness.

### Empirical Study of Approaches to Risk Mitigation

#### Overview

This study seeks to evaluate two approaches to risk mitigation when patients and consumers consult a CA for medical information by influencing their intent to act on the information they receive without first consulting a health care provider. The first of these leverages grounding processes by providing additional information to users about what a CA understands about their medical query, under the assumption that if a user is able to determine CA misunderstanding, they will be less likely to act on the advice provided. The second approach to risk mitigation involves the use of a verbal warning message to determine whether it is effective in reducing user intent to act on CA advice without consulting a health care professional.

#### Mitigation Approach #1: Risk Mitigation Through Improved Grounding

The purpose of the conversations with our voice-only CAs was to provide information about the use of medications under particular circumstances and to understand the effects of imperfect information exchanges in this space. Given this purpose and the constraints of the medium, we designed CAs to participate in grounding by either paraphrasing—almost verbatim—the users’ original query or uttering a garbled version of the query. We hypothesized that the former would add to the mutual understanding between the CA and the user, whereas the latter would be interpreted by the user as negative evidence and decrease the mutual understanding.

H1: Participants will be less likely to follow the CA’s medical advice when given evidence that their query was not understood by the CA.

#### Mitigation Approach #2: Risk Mitigation Through Disclaimer Warning

Ruiter et al [37] reviewed the literature on warning messages that elicit fear to promote precautionary motivation and self-protective action and found that moderate levels of fear result in maximum persuasion. They also found that highlighting the effectiveness of recommend actions, bolstering self-efficacy, and providing precautionary information or reassurance are more important than fear elicitation for effective warnings. Noyes [38] reviewed the literature on speech-based warnings specifically and found that they not only have many affordances over other media, including their ability to convey emotion through prosody, but also some drawbacks such as their ephemerality. Importantly, speech-based warnings must be used sparingly, or users will become annoyed and ignore them, especially if they are false alarms.

H2: Participants will be more likely to say that they will consult a physician before acting on medical advice provided by the CA when the advice is accompanied by a warning message that they should not act on the advice instead of talking to a physician.

## Methods

### Empirical Study

We conducted an empirical study to evaluate the effectiveness of these two approaches to risk mitigation when using CAs for medical information, performing a counterbalanced 2×2 factorial within-subjects experiment to evaluate our hypotheses. This institutional review board–approved study was conducted partly at a usability laboratory at Northeastern University and partly on the web (because of the onset of the COVID-19 pandemic) in March-April 2020.

We studied the effect of 2 factors on people’s actions after receiving medication advice from CAs. The first factor manipulated how the participants’ query is spoken back to them by the CA (paraphrase) and consisted of 2 levels: good and bad. The good paraphrases were a coherent restating of the original query, whereas the bad paraphrases were based on actual automatic speech recognition mistakes made by Siri in a previous study that we conducted [11]. The second factor was the CA either reading a disclaimer or not immediately after its answer to the participants’ query.

### Recruitment

Participants were recruited from a web-based job posting site and were eligible if they were aged 21 years or older and were native speakers of English (an earlier pilot had indicated that commercial CAs have extremely high misrecognition rates for nonnative speakers [11]). There were no other eligibility requirements. Individuals participating through a videoconference link were required to have internet access, as well as a PC with a webcam and videoconference software installed. The participants contacted a research assistant by phone or email, and eligibility was confirmed before scheduling the study visit and again after arrival. The participants were compensated for their time.

### Participants

A total of 32 participants completed the study. They were on average aged 42.44 (SD 14.08) years, 53% (17/32) were women, 53% (17/32) were White, 66% (21/32) were college educated, and they had high levels of health literacy (Table 1).

**Table 1:** Descriptive statistics of the study sample (N=32).

Characteristics		Values
Age (years), mean (SD)		42.44 (14.08)
Sex (female), n (%)		17 (53)
**Race, n (%)**		
	White	17 (53)
	African American	8 (25)
	Asian	2 (6)
	Other	5 (16)
**Education, n (%)**		
	High school	2 (6)
	Some college	6 (19)
	Technical school	3 (9)
	College graduate	13 (41)
	Advanced degree	8 (25)
**Experience with computers, n (%)**		
	Use one regularly	22 (69)
	Expert	9 (28)
	Other	1 (3)
**Health literacy (REALM^a^), n (%)**		
	7th-8th grade	2 (6)
	≥9th grade (*Adequate*)	30 (94)

^a^REALM: Rapid Estimate of Adult Literacy in Medicine.

### Conversational Assistant Apparatus

**Figure 1:**
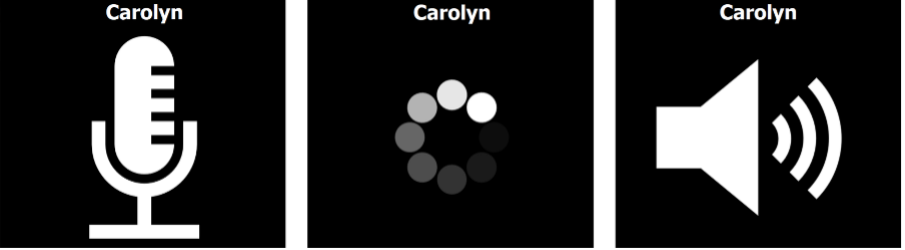
Icons used to indicate the conversational assistant state in the user interface.

Our study was designed to determine participant reactions to the planned manipulations. To achieve this in a controlled manner, the participants were asked to read queries verbatim to a simulated CA, and the CA responses were generated using a *Wizard of Oz* design, where the CAs were controlled by a research assistant. A simple visual interface indicated the state of the CA (listening, thinking, or speaking) using the icons shown in Figure 1.

Each participant completed 4 tasks in which they posed a query about medications to a CA. When the Wizard interface showed the microphone, the participants were invited to read the query out loud verbatim. Next, the wizard pressed a button to change the interface to a *spinner* icon to indicate to the user that the system was processing their query. Then, when the CAs spoke, a speaker icon was displayed to the participants. Each task had a different CA with a unique name and different synthesized voice. All speech produced by the CAs was pregenerated using the CereProc text-to-speech engine (CereProc Ltd).

### Task Scenarios

We used the scenarios and CA responses from a previous study that we had conducted to create our tasks [11] (Table 2). For each task, a participant asked the CA for advice about medications. The CA paraphrased the participant’s query and then offered advice. The advice was designed so that it would be considered harmful if acted on, given the context of each task (as judged by clinicians in the study by Bickmore et al [11]). The queries, paraphrases, and CA advice were all taken verbatim from the study by Bickmore et al [11], as spoken by the study participants or commercial CAs (Siri, Alexa, or Google Home).

**Table 2:** Tasks and CA responses used in this study.

	Task (query)	Good paraphrase	Bad paraphrase	CA^a^ advice
T^b^1	I am allergic to nuts, have asthma, and am taking a blood thinner for atrial fibrillation. I have a headache, what should I take for it?	You said you have a headache and want to know what to take. You are allergic to nuts, have asthma, and are taking a blood thinner.	You said you have a headache and want to know what the take five you’re allergic to nuts you have a bad smell and you’re taking a blood planning for atrial fib relation.	Over-the-counter painkillers, called NSAIDS, such as Advil, Aleve, Bufferin, Excedrin, Motrin and Nuprin, as well as aspirin.
T2	I am already taking Lisinopril for my blood pressure, and an antacid for my acid reflux. What nasal decongestant should I take for seasonal allergies?	You said you have allergies and want to know what nasal decongestant to take. You are taking Lisinopril, and an antacid for acid reflux.	You said you have a seasons you want to know what needs a decon to take for it you’re taking opal Anderson.	Sudafed is a class of decongestants. Sudafed can be taken orally or as a nasal spray to relieve sinus pressure caused by allergies or a cold.
T3	I have a soy allergy. Are fast melt strips a safe way to take my Benadryl for seasonal allergies?	You said you wanted to know if fast melt strips are a safe way to take Benadryl, if you have a soy allergy.	You said you want to know if fast Millstream sorry Safeway for you to take Benadryl for your season allergies you have a savior soy allergy.	The active ingredient in Benadryl Allergy Quick Dissolve Strips is an antihistamine used to relieve symptoms of allergy, hay fever, and the common cold.
T4	I am currently taking coumadin. Is Chinese ginseng root safe to take to improve my immune system?	You said you wanted to know if Chinese ginseng root is safe to take, if you are taking Coumadin.	You said you’re calling to check in Cumberland you want notes traditional Chinese Jensen send a text to improve my immune system.	Ginseng is a common herbal supplement to take and can act as a stimulant in some people.

^a^CA: conversational assistant.

^b^T: task.

### Measures

In addition to sociodemographic measures, health literacy was assessed using the Rapid Estimate of Adult Literacy in Medicine [39], and computer literacy was assessed using the single-item self-report measure, “How much experience do you have using computers?”, with responses ranging from “I’ve never used one” to “Expert.”

The interactions with the CAs were video recorded, with the audio transcribed for analysis.

After each task was completed, the participants were asked 3 questions:

1. *Action*: “Given this situation and the agent’s response, what would you do?” The participants’ open-ended responses were recorded.
2. *Likelihood*: “How likely are you to do that?” (scale anchors 1=*Not likely at all*, 4=*Not sure*, and 7=*Very likely*)
3. *Understanding*: “How well do you feel like the agent understood you?” (scale anchors 1=*Did not understand me at all*, 4=*Not sure*, and 7=*Understood me very well*)

After interacting with all 4 CAs, the participants were asked which of the CAs they would prefer to have future conversations with about medications. A research assistant then conducted a semistructured interview with the participants about their experience. During the interviews, the participants were asked to describe the 4 CAs and discuss how conversational grounding and the use of disclaimers affected their confidence in the CA as an assistive medical device. The interviews were audio recorded and transcribed for analysis.

### Procedure

Each participant took part in a single 60-minute usability session. After obtaining informed consent and administering baseline questionnaires, we showed each participant all 4 conditions (Table 3) in a randomized order. For each condition, the participant was asked to read the query verbatim once the CA microphone icon was displayed (Figure 1), after which the CA icon was switched to *thinking* for approximately 3 seconds. Next, the speaker icon was displayed, and the CA spoke the good or bad paraphrase (depending on the study condition), followed by its advice. Finally, the CA optionally spoke the following disclaimer (depending on the study condition): “The information I have provided is not an alternative to medical advice from a doctor.” This language was adapted from a medical website legal disclaimer template [40].

**Table 3:** Study conditions.

Condition	Paraphrase	Disclaimer
C1	Good	No
C2	Bad	No
C3	Good	Yes
C4	Bad	Yes

### Analysis

A total of 16 sessions were conducted at a usability laboratory, and 16 additional sessions were conducted through a videoconference link. The only difference between these 2 groups on baseline measures was that the median education level was significantly higher for those who participated over the videoconference link than for the laboratory participants (4 vs 3.5; W_1_=1280; P<.001). We therefore included education level as a covariate in our analyses. Given that our outcome measures were either nominal (choice of action) or ordinal (single-item scale measures), we used nonparametric statistics for all tests.

Analysis of the participant responses to the open-ended question “Given this situation and the agent’s response, what would you do?” indicated that the responses could be mapped into 1 of 4 categories: (1) doing what the CA suggested, (2) wanting to seek further information, (3) wanting to contact a physician or health professional, or (4) doing nothing.

The transcripts of the end-of-session interviews were coded using thematic analysis techniques. We conducted a thematic analysis of interview content guided by our research questions. The interviews were coded using NVivo software, version 12.5.0 (QSR International). Using open coding, we labeled discrete chunks of data. Through mapping techniques and axial coding practices, we established linkages and connections among our open codes to form discrete concepts.

## Results

### Principal Findings

We found that the bad paraphrase made the participants feel that the CA understood them less, showing that the manipulation in our study was successful. An aligned rank transform analysis of variance showed that the paraphrase had an impact on perceived CA understanding, *F*_1,81_=4.99; *P*<.001. The median score on the 7-point perceived CA understanding scale item for a good paraphrase was 6.5 compared with 4 for a bad paraphrase.

**Figure 2:**
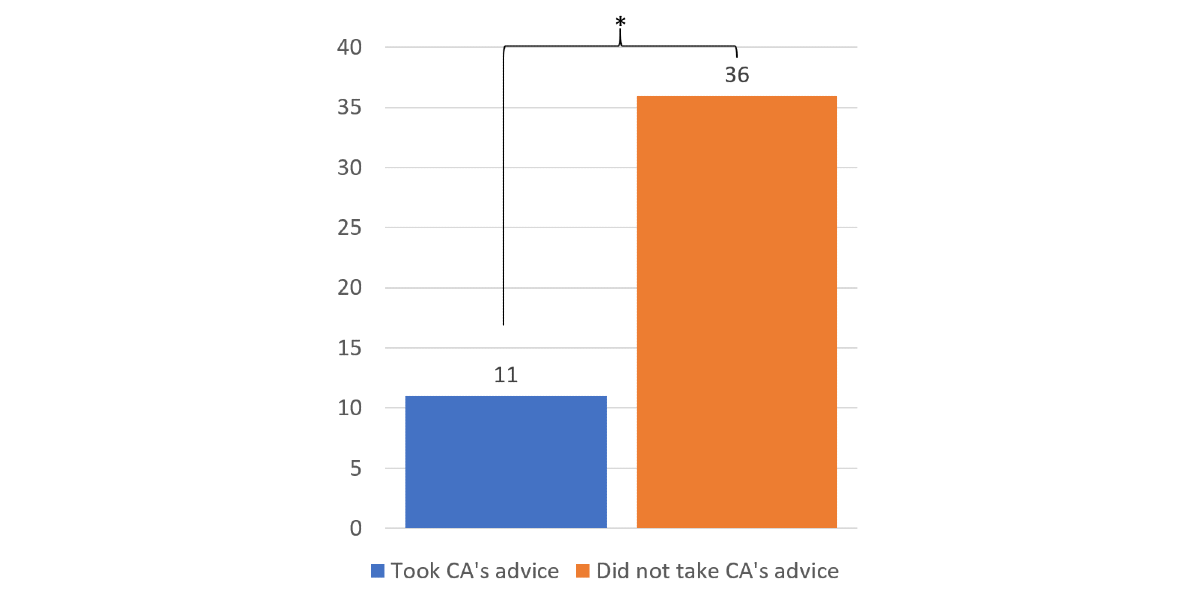
Feeling misunderstood by the CA resulted in fewer cases of taking its advice than feeling understood. CA: conversational assistant; *P=.002.

The participants who felt that the CA had not understood them, that is, those scoring below 4 on the perceived CA understanding scale, were less likely to take the CA’s advice than those who felt that the CA understood them, χ^2^_1_=8.81; *P*=.002. When the participants did not understand the CA, there were 36 cases of not taking the advice compared with 11 cases where advice was taken (Figure 2).

**Figure 3:**
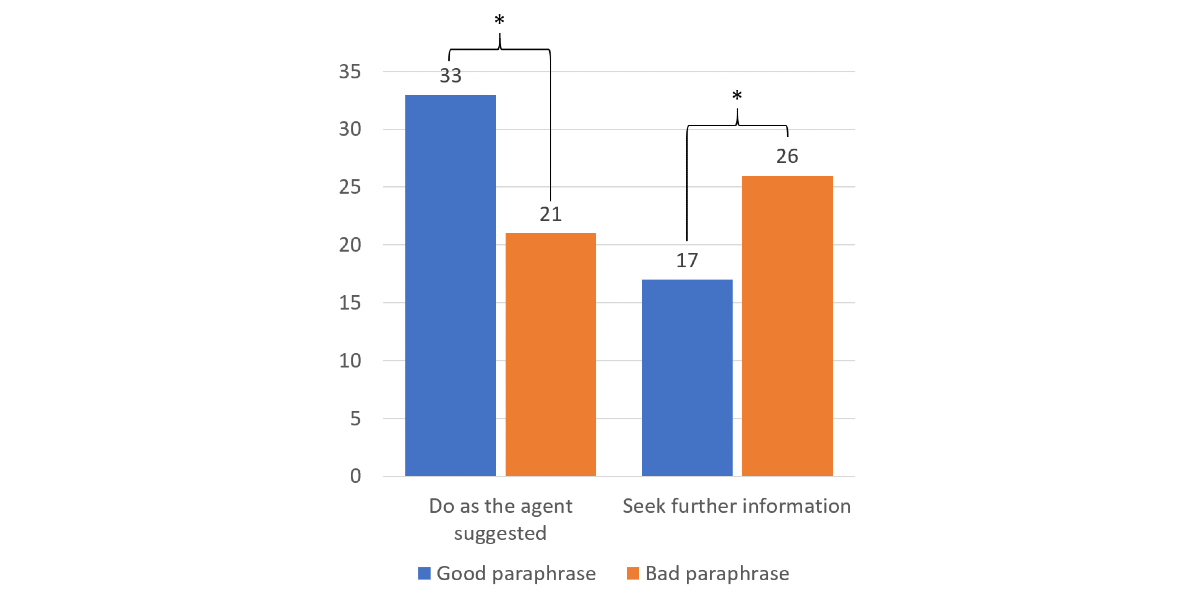
The number of cases of doing as the CA suggested or wanting to seek further information differed depending on the CAs’ paraphrase. CA: conversational assistant; *P=.04.

**Figure 4:**
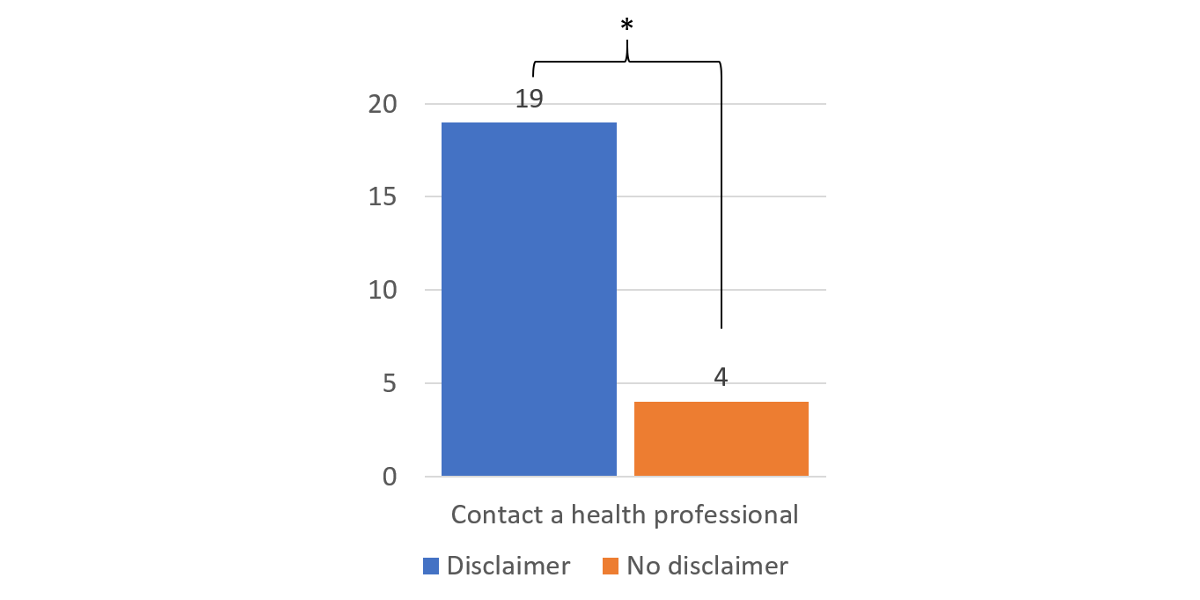
Having a disclaimer led more participants to consider contacting a health professional about the medications than when there was no disclaimer. *P=.001.

Of the 128 trials (4 per participant), there were 54 cases (42.2%) of the participants choosing to take the CA’s bad advice across all conditions (Table 4), and we found that the paraphrase significantly affected this choice, χ^2^_1_=3.1; *P*=.04 (Figure 3). Of the 54 cases, 33 (61%) occurred after a good paraphrase and 21 (39%) after a bad paraphrase. Across all 128 conditions, there were 43 (33.6%) cases of the participants wanting to seek more information, and the paraphrase factor also significantly affected this choice, χ^2^_1_=13.26; *P*=.04 (Figure 3). Of the 43 cases, 26 (60%) occurred after a bad paraphrase and 17 (40%) after a good paraphrase. Of the 128 cases, in 23 (18%), the participants said that they would contact a physician or health professional, and the disclaimer factor had a significant effect on this choice, χ^2^_1_=43.5; *P*=.001. Of these 23 cases, 19 (83%) occurred after a disclaimer and 4 (17%) occurred when there was no disclaimer (Figure 4).

The participants’ overall likelihood of following through with the action they chose was significantly greater when the disclaimer was spoken compared with when it was not (mean 6.64, SD 0.82 vs mean 6.38, SD 0.87), *F*_1,81_=9.1; *P*=.008. In addition, the overall likelihood of the participants wanting to seek further information about the medications and their side effects was significantly higher than the likelihood of contacting a physician or health professional (mean 6.79, SD 0.64 vs mean 6.2, SD 0.91), *F*_3,93_=5.02; *P*=.003.

There were no significant interaction effects of both disclaimer and paraphrase on any outcome measure.

**Figure 5:**
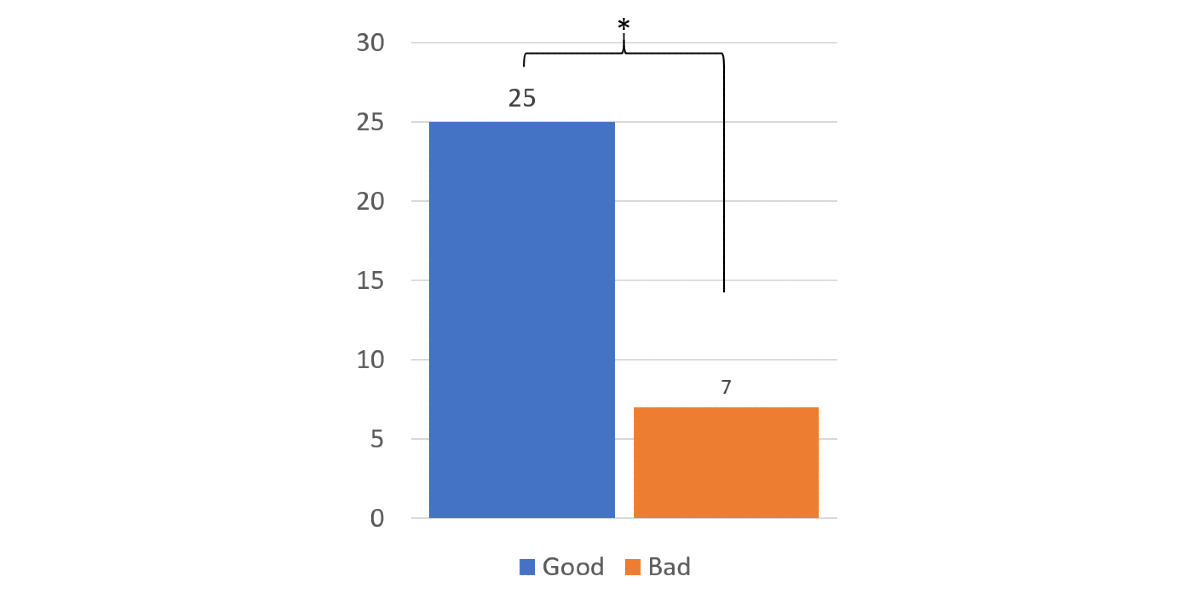
Participants chose a CA that paraphrased well more often than one that paraphrased poorly. CA: conversational assistant; *P=.001.

There was a significant difference among the conditions regarding the participants’ choice of CA for a future conversation about medications, χ^2^_3_=10.4; *P*=.01. Specifically, the number of cases where the participants chose to talk again with a CA that gave a good paraphrase (25/32, 78%) was significantly greater than the number of cases of participants wanting to talk again with the CA that paraphrased poorly (7/32, 22%), χ^2^_1_=10.12; *P*=.001 (Figure 5). There were no significant differences in preferences between the CAs that spoke the disclaimer and those that did not, χ^2^_1_=0.5; *P*=.47.

**Table 4:** The frequency of actions that the participants said that they would take after interactions with the Conversational Assistants (N=128).

Action	Number of participants who endorsed, n (%)
Do as agent suggested	54 (42.2)
Seek further information	43 (33.6)
Contact a health professional	23 (18)
Do nothing	8 (6.2)

### Qualitative Findings

#### Overview

The transcribed interviews resulted in a total of 145 minutes of audio files and 116 pages of transcription. Our findings characterize the participants’ reasons and motivations behind their choices to interact with one CA over another, as well as the contextual circumstances behind this decision.

Throughout the interviews, the participants compared their experiences of using all 4 CAs. Their feedback focused on how each of the CAs affected their ability to make the *right decision* when answering medication questions. During the interviews, the participants focused on the important elements required to make the right decision, such as their assessment of (1) the CA’s credibility, (2) the CA’s informational accuracy, and (3) the CA’s efficiency in the context of answering a medical query or question. Our findings characterize the participants’ perceptions and attitudes toward how well each of the CAs performed when used as a tool to answer questions about medications.

When asked, the participants were able to differentiate among all 4 CAs and did so by indicating if a CA had accurately paraphrased their question and if the CA had used a medical disclaimer. Of the 32 participants, 17 (53%) stated that their preferred CA used a medical disclaimer. Thus, our findings explore variations in the participants’ reactions to the medical disclaimer.

#### Disclaimers: Building CA Credibility by Establishing Fallibility

For some participants, the disclaimers added to the CA’s credibility by fulfilling a mental model aligned with their expectations similar to their expectations of medical websites and medical fitness devices (eg, Apple Watch and Fitbit). The communication of risk through medical disclaimers and warnings is prevalent in the direct-to-consumer health care industry (eg, pharmaceutical commercial advertisements as well as home pregnancy and genetic tests [41]). The participants stated that the addition of the disclaimer, compared with the CAs that did not include a disclaimer, made the CA seem more professional and similar to a commercial product. A participant stated as follows:

I like the disclaimer a lot. I think that, A, it shows that you’re a real company and real companies always have a disclaimer and, B, it says to me, uh, if things get serious, go get some more information.

P31

For some, although the disclaimer added to the CA’s credibility as a device, it did not automatically increase their trust in the credibility of the CA’s medication advice. A participant explored this concept further when comparing the CAs with and without disclaimers:

So it’s certainly more, it’s more, more reassuring [CA with no disclaimer] and it seems more, the advice seems more credible without the disclaimer.

P24

Most participants shared the view that the CAs who used the disclaimer reminded them not to automatically follow the advice without question. A participant echoed this sentiment:

It’s [CA with disclaimer] like okay, do your homework, you do more research. Don’t just, like, take my word as Gospel.

P19

The participants appreciated the reminder that the CA is not a replacement for the advice of a medical practitioner:

I think it’s responsible that they put it there. Because sometimes people jump to conclusions and then they get even more sick because they don’t really know what they are doing. It’s important to have that [disclaimer]. When they [the CA] were like “consult your doctor,” I was like okay well maybe I might need to.

P19

A participant expanded this view and explored how using a medical disclaimer may potentially help limit the spread of misinformation through its precautionary message:

I felt secured like...in this day of social media there’s so much misinformation out there. I have some coworkers that believe in those...conspiracy theories. So like if other people were to use this...device people would take it to heart. So I think it’s really important that we have...that disclaimers [are] added at the end.

P29

Throughout the interviews, the participants expressed that the medical disclaimers increased their confidence in the CA, their sense of safety, their trust that the CA was a viable medication assistant, and influenced them to reconsider the CA’s incorrect advice. However, these feelings were not shared by all participants. Our findings further reveal nuances reflected in the data with regard to the CAs’ use of medical disclaimers and warnings*.*

#### Disclaimers: Creating Confusion and Redundancy

For some, the use of the verbal disclaimer was superfluous. A participant described the warning as redundant:

I felt like it [the disclaimer] was kind of stating the obvious to be honest...you would probably expect that that’s not coming from a medical professional. So if you are asking that question, you’re kind of already accepting that.

P26

This comment demonstrated that, for some users, disclaimers do not communicate novel information but instead what they perceive as obvious information: the CA is not a clinician. In addition, although we incorporated the disclaimer as a risk communication strategy to increase user safety, some users expressed that the inclusion of the disclaimer ultimately communicated that the designers were concerned with avoiding potential legal liability:

I think the disclaimer is just...CY. Cover Yourself. You have to say that.

P23

Such a viewpoint can ultimately diminish a user’s perception of risk as well as the effectiveness of the precautionary warning and negatively affect the user’s trust in the device.

Other participants pointed out that the warning appended to the advice increased the length of the auditory information considerably. A participant explored these drawbacks when she stated as follows:

But in terms of how I process the information, um, that it was giving me, it just added on to the amount of information, and it kind of made it more confusing.

P21

Several participants agreed that when using a conversational system that relies on processing and understanding auditory information alone, the disclaimer obfuscated the CA’s answer, making the exchange inefficient.

Beyond inefficiency, the participants also expressed sensitivity toward warning fatigue. They stated that if a disclaimer were used during every interaction, they would stop taking the warning seriously and discontinue their use of the CA entirely, reflecting prior findings on speech-based warnings [38]. A participant succinctly stated as follows:

[The disclaimer] makes me feel like why am I wasting my time with this [CA] when I should be going to a real professional?

P29

By reminding the user that the system was not a replacement for the advice of a medical practitioner, some participants not only reconsidered the accuracy of the advice, but they also determined that the CA’s functionality as a medication assistant was limited.

#### Paraphrasing: Developing Trust and Facilitating Confidence

When describing the properties related to making an informed medical decision, the participants explored how trusting the information source is critical. They spoke of trusting the CA that demonstrated conversational understanding. A participant described how grounding affected her perceptions of the system:

She [good paraphrase CA]...was geared exactly to what I was asking, um, and yeah...she just gave me the most confidence in, in the answer that I received.

P19

The participants compared CAs that accurately or inaccurately paraphrased their questions. They reported that an inaccurate paraphrase decreased the likelihood that they would follow the CA’s advice. A participant stated as follows:

She [bad paraphrase CA] just didn’t really understand what I was asking, so I felt uneasy about the information.

P20

Mistakes such as a bad paraphrase at the beginning of the interaction lowered users’ confidence in the CA’s abilities, causing users to immediately question the soundness of the CA’s advice. A participant described how quickly a bad paraphrase creates doubt:

But for the bad paraphrasing, like right away when talking, you just know that they’re providing me wrong information right away.

P29

However, when the CA paraphrased the participant’s query correctly, the participants reported higher confidence in the accuracy of the CA’s incorrect advice. A participant emphatically stated as follows:

Oh, absolutely, [good paraphrase] is number one. The trust comes right there. I said “this.” You [the CA] listened.

P23

From the participants’ perspective, the CA’s use of a good paraphrase not only demonstrated a certain level of conversational understanding, but was also perceived as a meaningful and responsive component of the conversational exchange. In comparison, the disclaimer was described as a tacked-on *canned* statement. From the participants’ perspective, the disclaimer would be present in the conversation irrespective of what the participant actually said, and the disclaimer's overall contribution to the system was mainly as a functional safety alert.

## Discussion

### Principal Findings

We found that grounding the feedback provided by a CA, in the form of paraphrases of user input, was effective at decreasing potentially harmful actions by the participants when the feedback indicated that the CA did not fully understand their query, supporting H1. We also found that signaling a lack of understanding significantly increased the likelihood that the participants would seek additional information before acting on the CA’s recommendations. A warning message that the CA’s advice should not be taken as an alternative to medical advice from a physician was effective at increasing the likelihood that the participants would consult a physician before acting on the CA’s advice, supporting H2.

When interviewed, several of the participants indicated that disclaimers had benefits beyond promoting safe behavior, for example, by increasing the credibility of the device and their sense of reassurance and security in using it. However, several participants also indicated that disclaimers should be used sparingly and kept as brief as possible to avoid obfuscating the CA’s response by adding content in the limited audio channel.

Grounding, in the form of paraphrasing participant queries, was cited as being important in assessments of credibility and trust, at least when the grounding indicated that the CA had properly understood a query. Incorrect paraphrases not only led to a decreased likelihood of acting on the CA’s advice, but also affected negatively the participant’s assessment of the CA and desire to use it in the future.

Our quantitative and qualitative findings demonstrate that accurate grounding increased the participants’ confidence in the CA’s medical advice by signaling that the user was properly heard. In this experiment, all our CAs relayed harmful medication advice. As a result, grounding alone was insufficient for mitigating user risk and potentially could have misled the participants to act on harmful medication advice. A participant described their response to a good paraphrase CA that did not incorporate a disclaimer as follows:

I felt like she did understand the full scope of the question and then was subsequently able to answer it by saying that it was safe to take.

P32

When asked directly for their perceptions of the CA that used a disclaimer, this participant explored how the addition of a disclaimer could keep users safe:

I would say…[the disclaimer] can reinforce that if you’re not entirely sure or in the instance where maybe…you are impulsively doing something, that reinforcement that maybe you do need to seek another opinion could sway you from doing something that maybe you should or shouldn’t do.

P32

### Limitations

Our study includes several limitations, including the small convenience sample used. Restricting eligibility to native speakers of English certainly skewed our sample, but based on pilot testing, CA sessions with nonnative speakers yielded insufficient data, given the extremely high nonrecognition rates. Limiting participants to scripted utterances decreases the validity of our findings compared with allowing them to query CAs in their own words. However, we were primarily interested in investigating user perceptions of mitigation strategies and feel that our controlled examples achieved that by using actual unconstrained participant queries and actual CA responses from a prior study. We did not assess the participants’ prior knowledge of the specific medical topics that we used as examples in our study, and this could have biased our findings. Finally, CA trust, credibility, and warning fatigue change over time and must ultimately be assessed in a longitudinal context. For example, some researchers have found that a user’s familiarity with a product significantly decreases their tendency to attend to warnings [42,43], indicating that warnings may lose their effectiveness over time with regular product use. Our study examines only first impressions of the mitigation strategies evaluated.

### Conclusions

Designers of conversational systems should consider incorporating both warning messages and grounding techniques in response to user medical queries where harm could occur if consumers act on the advice, whether it is correct or not. To decrease alarm fatigue, warnings should be used sparingly and only when a CA determines that the user is trying to obtain actionable medical advice. In contrast, grounding feedback should always be provided because it has utility for all kinds of queries, both medical and nonmedical.

Note that use of these techniques does not guarantee safety: a CA may fully understand the user’s query and provide grounding evidence of its understanding, but it may still retrieve incorrect advice or the user may misunderstand it [11]. In these cases, grounding may actually result in misplaced trust and increased user intent to act on potentially harmful advice. Ultimately, to maximize safety, grounding should convey the CA’s understanding of what the user understands about the advice given, as well as the CA’s understanding of what the user plans to do with the requested information.

The potential for AI systems to cause harm has long been recognized [44,45], but CAs that provide advice through unconstrained natural language represent one of the most challenging types of systems to ensure safety for. There is now increased interest in addressing issues of bias, safety, and validity in black box AI natural language processing systems [46]. However, recently proposed approaches that focus on describing appropriate contexts of use or the use of validation test suites [19] cannot possibly cover all cases that could lead to user harm, given the very large number of contextualized discourses that are possible. Despite the high error rates currently exhibited by CAs and with no clear approach to ensuring their safety, many experts feel that CAs will soon be able to provide reliable medical advice. A Delphi panel that comprised managers, physicians, researchers, and industry experts concluded that CAs will be able to provide “solid medical advice” within the next 5 years [47]. We feel that this projection is not based on an in-depth understanding of the issues, and that risk mitigation strategies such as those we have outlined here are needed until approaches to provably minimize the potential for CAs to give harmful advice are developed.

We reiterate the conclusions in the study by Bickmore et al [11] that unconstrained natural language input—typed text or speech—should not be used in CAs that are designed primarily to provide laypersons with medical advice. Such CAs have the potential to cause harm if users act on incorrect advice without first consulting a health care professional. Consumers lack mental models of CAs and cannot know the extent of the CAs’ medical expertise or their linguistic capabilities and, even with improved grounding, may fail to recognize when the CA does not properly understand their communicative intent, fail to recognize when the CA has retrieved incorrect information, or fail to properly understand the CA’s advice. The 2 mitigation strategies that we have explored in this work should only be used on CAs intended for other purposes (eg, general use, such as Siri or Alexa) when users naively ask for medical advice.

### Future Work

The development of AI models that are explainable is an active area of research that is highly relevant to the implementation of safe CAs [48]. Indeed, explanation of how a CA understands a user can be seen as grounding, and the development of these methods targeting layperson understanding is an important direction of investigation. Given the complexity of the underlying AI models used in state-of-the-art CAs, additional media beyond the voice channel may be required to provide users with a fuller understanding of what is behind CA advice.

Identification of potentially unsafe user queries is a prerequisite for delivering targeted warning messages and also represents an important area of research. Such identification is nontrivial, given the many potential contexts of use, user states, and user intents, but is an important area of research in its own right. Persistent knowledge of users’ medical condition, medications, and other electronic health record information could be critical in the medical domain.

The interaction of disclaimers and grounding strategies should be further explored. For example, the presence or absence of disclaimers may affect how users engage with grounding strategies.

Longitudinal studies of user interactions with CAs are particularly important to assess changes in attitudes toward a CA and changes in reactions to warnings over time.

Establishing the prevalence of actual harm from incorrect medical advice from a CA would be important to further motivate this area of research, requiring large-scale epidemiological surveys of patients, consumers, and medical professionals.

Finally, there may be many additional mitigation strategies that could be explored, such as having a CA engage users in dialog to understand why they are asking for medical advice and what they intend to do with the information provided before any advice is provided.

## Abbreviations

AI: artificial intelligence
CA: conversational assistant
ECA: embodied conversational agent
TAM: Technology Acceptance Model
